# Astrocytes in Neuropathologies Affecting the Frontal Cortex

**DOI:** 10.3389/fncel.2019.00044

**Published:** 2019-02-12

**Authors:** Ulla-Kaisa Peteri, Mikael Niukkanen, Maija L. Castrén

**Affiliations:** Department of Physiology, Faculty of Medicine, University of Helsinki, Helsinki, Finland

**Keywords:** astrocyte, frontal cortex, Alzheimer’s disease, Huntington’s disease, frontotemporal dementia, neurodegeneration, induced pluripotent stem cells

## Abstract

To an increasing extent, astrocytes are connected with various neuropathologies. Astrocytes comprise of a heterogeneous population of cells with region- and species-specific properties. The frontal cortex exhibits high levels of plasticity that is required for high cognitive functions and memory making this region especially susceptible to damage. Aberrations in the frontal cortex are involved with several cognitive disorders, including Alzheimer’s disease, Huntington’s disease and frontotemporal dementia. Human induced pluripotent stem cells (iPSCs) provide an alternative for disease modeling and offer possibilities for studies to investigate pathological mechanisms in a cell type-specific manner. Patient-specific iPSC-derived astrocytes have been shown to recapitulate several disease phenotypes. Addressing astrocyte heterogeneity may provide an improved understanding of the mechanisms underlying neurodegenerative diseases.

## Introduction

Astrocytes are implicated as active mediators of synaptic activity, synaptogenesis and neurogenesis and are crucial in maintaining extracellular homeostasis and controlling blood-brain barrier permeability (Zhao et al., 2015; Allen and Lyons, 2018; Marina et al., 2018). Considering their versatile role in regulating brain function, it is no surprise that astrocyte malfunctions have been connected to various neurodegenerative disorders (Phatnani and Maniatis, 2015). Astrocytes are known as a morphologically and functionally diverse population of cells that differ both between distinct brain regions as well as within specific areas (Vasile et al., 2017). This diversity is reflected in their pathological features observed in psychiatric disorders (Rajkowska et al., 2002; Wallingford et al., 2017). Drugs used to treat mood disorders affect astrocytes as well and antidepressant effect on neurons is considered to be partially due to induction of astrocytic release of trophic factors (Marathe et al., 2018). Combined astrocyte and neuron-mediated effects also influence responses of antipsychotics (Khan et al., 2001). However, typical and atypical antipsychotics may display differential effects on the gliotransmitter release and the inflammatory response of astrocytes (Tanahashi et al., 2012; Bobermin et al., 2018). Understanding the function of astrocytes is therefore crucial for disease modeling and for developing treatments. Heterogeneity of the cell population and species-specific differences pose a challenge in the study of astrocytes. A method developed by Takahashi et al. (2007) allows the reprogramming of somatic cells into iPSCs, which can be used to generate patient-specific cells of a desired type (Takahashi et al., 2007). In this review, we describe how iPSC-derived astrocytes have been used to model neurodegenerative disorders involving frontal lobe malfunctions.

## Astrocyte Heterogeneity in the Brain

The generation of cortical glia is initiated once neurogenesis has been completed. The temporal patterning is based on a positive feedback signal from new-born neurons (Molofsky and Deneen, 2015; Takouda et al., 2017). Astrocyte progenitors migrate radially, obtaining their region specific properties upon maturation and this process continues postnatally (Colombo et al., 1997; Tsai et al., 2012). Several genes enriched in neuronal progenitors are also expressed in astrocytes, suggesting that astrocytes retain some proliferative potential even in the mature brain (Cahoy et al., 2008). However, only a distinct subset of astrocytes show neurogenic potential (Ghashghaei et al., 2007; Bardehle et al., 2013).

Mature astrocytes can be distinguished based on their morphology and functional properties. In the human cortex, astrocytes are morphologically categorized into four subtypes; interlaminar, protoplasmic, varicose projections and fibrous astroglia, located in the layers I and II, III and IV, V and VI and in the white matter, respectively (Vasile et al., 2017). The brain also contains other, both morphologically and functionally, distinct astrocytes such as elongated radial glia-like tanycytes and unipolar BG with several radially ascending processes. Tanycytes specialized in the regulation of neuroendocrine functions are located in the hypothalamus (Prevot et al., 2018) while BG modulate the efficacy of the synaptic transmission of Purkinje cells in the cerebellum (De Zeeuw and Hoogland, 2015).

Some astrocyte subtypes found in the human cortex are not represented in the rodent brain. Furthermore, human cortical astrocytes exceed their mouse counterparts both in complexity and size, and propagate calcium signals several times faster (Oberheim et al., 2009). Although human and mouse astrocytes share similar properties related to their effects on synapse formation, they differ in their function and transcriptional profiles (Zhang et al., 2016). Species-specific functional differences in glial cells are supported by improved learning and memory in chimeric animals following engrafting human glia into mouse brain (Han et al., 2013).

A higher relative number of astrocytes in the human frontal cortex, compared to that of many other species including other primates, is thought to be due to the high metabolomic cost of maintaining a bigger brain size (Bass et al., 1971; Sherwood et al., 2006). One of the key mechanism’s astrocytes apply to provide energy to neurons is via the astrocyte-neuron lactate shuttle. This metabolomic coupling is known to be crucial for memory formation (Alberini et al., 2018). Astrocytes respond to neuronal activity with spatially and temporally regulated Ca^2+^ fluctuations that shape neuronal activity via the regulation of the gliotransmitter release (Semyanov, 2019). Molecular and functional variations in astrocytes are considered to contribute to differences in distinct neural circuit signaling (Chai et al., 2017; Morel et al., 2017; Xin and Bonci, 2018). Another central role of astrocytes is the regulation of neurotransmitter uptake including glutamate via excitatory amino acid transporters 1 and 2 (EAAT1, EAAT2, respectively) in humans, or glutamate/aspartate transporter (GLAST) and glutamate transporter-1 (GLT-1) in rodents (Roberts et al., 2014; Meunier et al., 2017). Regulation of the extracellular neurotransmitter levels is affected in a number of neuropsychiatric disorders (John et al., 2012).

## Astrocytes as Mediators of Pathologies Affecting the Frontal Cortex

The frontal cortex is responsible for higher executive functions such as cognition and working memory (Fuster, 2002). The expression of genes involved in processes mediating synaptic plasticity, memory and learning is, respectively, enriched in a human and primate frontal cortex (Sjostedt et al., 2015; Garcia-Cabezas et al., 2017). A high level of flexibility is necessary for learning and memory functions but may also lead to increased structural vulnerability, which may explain why aberrations in the frontal cortex are connected to several neuropathologies (John et al., 2012; Feresten et al., 2013; Torres-Platas et al., 2016).

Astrocytes contribute to the regulation of neuronal activity that is altered in several frontal cortex pathologies (Braun et al., 2009; Cao et al., 2013; Lima et al., 2014; Bull et al., 2015; Ebrahimi et al., 2016; Beamer et al., 2017). A common feature for brain diseases is the activation of astrocytes into an inflammatory, reactive state (Chanaday and Roth, 2016). White matter astrocytes in the frontal cortex appear to be especially vulnerable to ischemic stroke, leading to disrupted gliovascular interactions caused by astrogliosis (Chen et al., 2016). Astrogliosis in the frontal cortex, upon aging, is also linked to mood disorders (Miguel-Hidalgo et al., 2000; Narita et al., 2006). Neurodegenerative disorders have overlapping characteristics suggesting common underlying pathological mechanisms. For instance, CTE caused by repeated head injuries, displays a similar accumulation of neurofibrillary tangles to that in AD but can be differentiated from AD by astrocytic tangles that are considered a hallmark of CTE (Turner et al., 2016; Hsu et al., 2018). Below, examples of neurodegenerative disorders with fronto-temporal pathologies and studies employing iPSC-derived astrocytes are described.

### Alzheimer’s Disease

Alzheimer’s disease is a progressive neurodegenerative disease that manifests through cognitive impairment, motor abnormalities and behavioral changes. AD pathology is hallmarked by the accumulation of insoluble amyloid-β (Aβ) plaques, amyloid deposits in the blood vessel walls and aggregation of the microtubule protein tau, within neurons. The abnormalities seen in AD usually occur first in the frontotemporal region then spread progressively to other areas of the neocortex (Masters et al., 2015).

The contribution of astrocytes in AD pathology comprises of both the loss of neuroprotective features as well as the acquirement of pathological properties. Initially, astrocytes uptake and degrade Aβ and have a neuroprotective role. However, disease progression often leads to impaired astrocytic Aβ clearance and induces toxic gain-of-functions that contribute to disease progression (Garwood et al., 2017). Neural plaques adjacent to GFAP expressing astrocytes are known to induce hypertrophy and there is also evidence showing that astrocyte reactivity may precede plaque formation (Teneka et al., 2005; Olabarria et al., 2010; Rodrieguez-Veitez et al., 2015). Morphological aberrances in AD astrocytes that compromise vascular coverage are detrimental to neurovascular regulation, while disrupted potassium (K^+^) mediated neurovascular coupling, due to downregulation of K^+^ channels Kir4.1 and BK_Ca_, result in abnormal regional cerebral blood flow (Acosta et al., 2017).

Aβ has been shown to alter the expression of metabotropic glutamate receptor 5 (mGluR5) and nicotinic acetylcholine receptors (nAchRs) in astrocytes, which leads to changes in Ca^2+^ homeostasis and signaling (Haughey and Mattson, 2003; Xiu et al., 2005; Lim et al., 2013). Excitotoxicity is a common characteristic of AD and astrocytes contribute to excessive glutamate signaling. Insufficient clearance of glutamate is connected to the reduced expression of glutamate transporters and their aberrant trafficking, which has been linked to altered cholesterol synthesis (Masliah et al., 1996; Tian et al., 2010; Merlini et al., 2011; Talantova et al., 2013). Furthermore, the release of glutamate has been shown to be enhanced in AD astrocytes (Talantova et al., 2013).

Reactivity is a common feature of AD astrocytes. Aβ induces the astrocytic release of pro-inflammatory mediators and, in turn, pro-inflammatory signals stimulate astrocytic Aβ production leading to a positive feedback loop between astrocyte Aβ response and production (Gonzáles-Reyes et al., 2017). S100β-positive astrocytes are connected to AD pathology and they are reduced following immunization against Aβ (Neus Bosch et al., 2015). S100β expressed by astrocytes is important for the regulation of neuronal oscillations associated with cognitive flexibility and depressive behavior (Stroth and Svenningsson, 2015; Brockett et al., 2018).

Glucose hypometabolism can precede clinical symptoms of AD (Mosconi et al., 2006). There is evidence that carriers of apolipoprotein E𝜀4 (APOE𝜀4) allele, with an increased risk for AD, have lower levels of glucose metabolism in various brain regions, including the prefrontal cortex, before the manifestation of clinical symptoms (Reiman et al., 2004). Dementia in AD is related to altered lactate processing. Under normal circumstances lactate-producing enzymes are down-regulated with age and an increase in the expression of these enzymes improve memory in wild type mice but leads to memory deficits in AD mice (Harris et al., 2016). Astrocyte defects in AD have been described extensively in a recent review (Acosta et al., 2017).

### Huntington’s Disease

Huntington’s disease (HD) is characterized by motor dysfunction, cognitive impairment and neuropsychiatric features. HD is an inherited neurological disorder caused by CAG trinucleotide repeat expansion in the gene encoding Htt. The expansion gives rise to a mutated form of Htt (mHtt) with an abnormally long polyglutamine sequence which leads to the formation of mHtt aggregates (Bates et al., 2015). Clearance of aggregates is more efficient from astrocytes than from neurons, rendering astrocytes more resistant to mHtt accumulation (Zhao et al., 2016; Jansen et al., 2017; Zhao T. et al., 2017). Eventual accumulation of mHtt into astrocytes results in altered glutamate homeostasis and, sub-sequentially, neuronal excitotoxicity (Shin et al., 2005; Bradford et al., 2009). In addition to the enhanced release of glutamate, the presence of mHtt in astrocytes decreases the expression of glutamate transporters in an age-dependent manner (Lievens et al., 2001; Estrada-Sanchez et al., 2009; Faideau et al., 2010; Lee et al., 2013). However, excitotoxicity in HD neurons has also been reported without defects in the glutamate clearance (Parsons et al., 2016).

Huntington’s disease astrocytes possess an altered K^+^ signaling due to the decreased expression of Kir4.1 (Tong et al., 2014; Zhang et al., 2018). Restoration of Kir4.1 function can ameliorate impaired GLT1-mediated homeostasis and, sub-sequentially astrocyte Ca^2+^ signaling, implying a causative effect of Kir4.1 dysfunction on these mechanisms (Tong et al., 2014; Jiang et al., 2016). Kir4.1 defects precede the appearance of reactive astrocytes, indicating that inflammation is a secondary effect of HD pathology possibly induced by neurotoxicity (Tong et al., 2014).

Both reactive astrocytes and microglia have been implicated in the pathogenesis of HD (Khakh et al., 2017). Microglia promote the reactivity of astrocytes via the secretion of pro-inflammatory factors such as Il-1α, TNFα, and C1q (Liddelow et al., 2017). Reactive astrocytes have an impaired ability for synaptic maintenance and decreased phagocytic capacity (Bradford et al., 2009; Haim et al., 2015). Additionally, they promote degeneration of a subset of neurons and mature oligodendrocytes (Liddelow et al., 2017). Activation of the JAK/STAT3 pathway appears to be a common pathological feature of HD and AD. Astrocyte specific inhibition of this pathway, in animal models, reduces the reactive astrocyte phenotype (Haim et al., 2015). Interestingly, some studies have shown that reactive astrocytes can also have a neuroprotective role in HD (Haim et al., 2015; Liddelow et al., 2017).

Accumulation of mHtt disrupts exosome secretion from astrocytes (Hong et al., 2017). This can be connected to the reduced BDNF release from astrocytes (Hong et al., 2016). BDNF signaling is associated with HD pathogenesis and restoration of BDNF release from astrocytes has been shown to have neuroprotective effects (Giralt et al., 2010; Hong et al., 2016; Reick et al., 2016). However, there is also evidence that at early stages of HD, TrkB signaling is altered due to an indirect effect of p75 neurotrophic receptor (p75^NTR^) activity, indicating that signaling defects may precede aberrant secretion of BDNF, a ligand of both TrkB and p75^NTR^ (Plotkin et al., 2014).

### Frontotemporal Dementia

Frontotemporal dementia (FTD) is an umbrella term for neurodegenerative diseases affecting the frontal or temporal lobes. Behavioral changes and deficits in executive functioning and language characterize FTD (Bang et al., 2015). The role of astrocytes in FTD is not fully understood. However, FTD pathology is known to involve astrogliosis that occurs at an early stage of the disease progression and precedes neuronal loss (Su et al., 2000; Kersaitis et al., 2004). Astrocytic degeneration is marked by the expression of apoptotic markers, such as caspase-3, and morphological changes (Su et al., 2000; Broe et al., 2004). Apoptotic astrocytes in FTD have been correlated with the degree of frontotemporal atrophy and significant astrogliosis has been observed to overlap with areas showing disturbed cerebral perfusion (Martinac et al., 2001; Broe et al., 2004). In theory, astrocyte degeneration could cause disruptions similar to those seen in AD and HD, but the possible role of astrocyte degeneration in FTD pathogenesis remains unclear (Su et al., 2000).

## Modeling Psychopathologies Using Human Cells

In recent years, a number of astrocyte differentiation methods have been developed and advances on the use of iPSC-derived astrocytes have been reviewed in a recent paper (Zheng et al., 2018). Below, the application of iPSC-derived astrocytes and their use to model frontal cortex defects are discussed. The studies represented are summarized in Table 1.

**Table 1:** Summary of studies on iPSC-derived astrocytes in modeling frontocortical pathologies.

Disease	Mutation	Astrocyte differentiation	Key findings	Reference
AD	*PSEN* (FAD) *APOE4*+/+ (SAD)	iPSC-derived NPC conversion to astroglia in the presence of CNTF, BMP2, FGF2, EGF.	Both SAD and FAD astrocyted exhibited reduced morphological heterogeneity, aberrant expression of S100β, and altered cytokine secretion. Altered EAAT1 distribution only seen in SAD.	Jones et al., 2017
AD	*PSEN1 ΔE9*	Expansion of NPCs in suspension culture in the presence of FGF2 and EGF. Astrocyte differentiation in the presence of CNTF and BMP4.	Astrocytes produce Aβ with aberrant uptake. Altered cytokine secretion, increased production of ROS. Induce aberrant Ca2+ signaling in healthy neurons.	Oksanen et al., 2017
AD	*APOE*^𝜀4/𝜀4^ *APOE*^𝜀3/𝜀3^	Neural induction in suspension culture followed by neural rosette formation and generation of NPCs. Astrocyte differentiation in the presence of CNTF, BMP4, and Heregulin-β.	ApoE isoforms have distinct properties with *APOE*^𝜀3/𝜀3^ astrocytes having greater neuroprotective and synaptogenetic potential.	Zhao J. et al., 2017
AD	*APOE4 APOE3*	NPCs generation in an adherent culture. Astrocyte differentiation in the presence of FGF2 and BMP4.	Aberrant production and uptake of Aβ. In 3D culture Aβ starts to accumulates in the organoids. Changes in the gene expressions related to lipid metabolism.	Lin et al., 2018
AD	APP-KO APPswe/swe APP V717F	NPCs were generated in an adherent culture. Astrocyte differentiation in a suspension culture in the presence of FBS and EGF.	Astrocytes have aberrant cholesterol metabolism. Lipoprotein and Aβ endocytosis are impaired.	Fong et al., 2018
HD	*Hit*	NPCs were generated in suspension in the presence of growth factors. Astrocyte differentiation was induced by plating the NPCs in the absence of FGF2.	Generated astrocytes exhibited enhanced cytoplasmic vacuolation under basal conditions.	Juopperi et al., 2012
HD	*Hit*	Astrocyte differentiation of iPSCs in neural differentiation medium in the presence of CNTF.	Blocking soluble TNFα suppresses pathological inflammatory response in astrocytes.	Hsiao et al., 2014
HD	*Hit*	Astrocyte differentiation of iPSCs in neural differentiation medium in the presence of CNTF.	Increased inflammatory response and expression of VEGF-A in HD-astrocytes lead to compromised vascular reactivity.	Hsiao et al., 2015
FTD/ALS	TDP-43 M337V	NPCs were cultured in the presence of LIF and EGF in followed by expansion with FGF2 and EGF. Terminal differentiation into astrocytes induced by growth factor withdrawal.	Astrocytes showed accumulation of cytoplasmic TDP-43 resulting in lowered cell survival.	Serio et al., 2013
FTD	*MAPT* N279K	NPC differentiation induced by lentiviral induction of SOX10 followed by treatment with SAG, PDGF, FGF2, NT3, IGF, and LDN. Astrocyte differentiation in the presence of IGF, CNTF, and dbcAMP	Astrocytes showed changes in *TAU* expression, hypertrophy, increased vulnerability to oxidative stress and altered transcriptomic profile. In co-culture system FTD astrocytes altered responses to oxidative stress in healthy neurons.	Hallmann et al., 2017


Defects in both the clearance and production of Aβ, associated with AD, can also be seen in iPSC-derived AD astrocytes and appear to involve aberrant lipid metabolism (Oksanen et al., 2017; Fong et al., 2018; Lin et al., 2018). When studying the effects of APOE genotype Lin et al. (2018) demonstrated that *APOE4* astrocytes show differences in the transcriptomic profile compared to isogenic *APOE3* cells, as well as a diminished ability in clearing Aβ (Lin et al., 2018). The role of ApoE in the Aβ clearance is still unresolved and some studies claim that ApoE is crucial for the degradation and removal of Aβ, while others have shown that ApoE promotes neurodegeneration (Holtzman et al., 1999; Koistinaho et al., 2004; Liao et al., 2014; Shi et al., 2017). In co-culture studies *APOE3* exhibited a greater ability to promote neuronal support and synaptogenesis (Zhao J. et al., 2017). Different properties of *APOE* isoforms in human astrocytes are in agreement with previous studies in mice (Wang et al., 2005). Jones et al. (2017) studied the function of AD astrocytes generated from iPSCs modeling early-onset FAD with mutation in *PSEN* and late-onset SAD with the *APOE4* genotype. Both FAD and SAD astrocytes showed reduced morphological heterogeneity and aberrant expression of S100β. However, altered distribution of EAAT1 was only seen in SAD astrocytes (Jones et al., 2017). Altered secretion of inflammatory cytokines was found in both FAD and SAD, as well as in astrocytes with the *PSEN1 ΔE9* genotype generated by Oksanen et al. (Jones et al., 2017; Oksanen et al., 2017). *PSEN1 ΔE9* astrocytes also displayed changes in Ca^2+^ homeostasis, mitochondrial metabolism, ROS production and lactate secretion, thus covering all classical features of AD pathology (Oksanen et al., 2017).

Inflammatory responses were studied by Hsiao et al. (2015) in iPSC-derived HD astrocytes and an increase in the expression of VEGF-A, with further up-regulation after inflammatory cytokine treatment, was found. This leads to the enhanced proliferation of endothelial cells and the compromised survival of pericytes. As a result, poor pericyte coverage of blood vessels cause vascular reactivity and disrupts the blood-brain-barrier (Hsiao et al., 2015). Additionally, they demonstrated that the TNFα inhibitor XPro1595 successfully suppressed the inflammatory responses both in human astrocytes as well as primary astrocytes propagated from the brain of a transgenic HD mouse model (R6/2) (Hsiao et al., 2014). Juopperi et al. (2012) showed that HD astrocytes display increased cytoplasmic vacuolization (Juopperi et al., 2012). This phenotype is also present in HD lymphoblasts (Nagata et al., 2004; Martinez-Vicente et al., 2010). The findings in iPSC-derived HD astrocytes are consistent with astrogliosis as a key characteristic of HD pathology.

Frontotemporal dementia astrocytes, derived from iPSCs with mutations in genes encoding microtubule-associated protein TAU (MAPT) and TDP-34, demonstrated an increased susceptibility to oxidative stress and compromised survival (Serio et al., 2013; Hallmann et al., 2017). In M337V *TDP-34* astrocytes, lowered survival paralleled the accumulation of TDP-43 (Serio et al., 2013). This phenomenon has been implicated in astrocyte dysfunction in CTE (Jayakumar et al., 2017). In N279K *MAPT* astrocytes, the expression of 4R-TAU isoform was increased as reported in FTD patients (Ghetti et al., 2015; Hallmann et al., 2017). N279K *MAPT* astrocytes displayed morphological changes and increased GFAP expression, usually linked to reactivity, as well as altered gene expression profiles. In co-culture assays with healthy neurons, N279K *MAPT* astrocytes increased the vulnerability of neurons to oxidative stress (Hallmann et al., 2017). However, M337V *TDP-34* astrocytes did not exert toxic effects on neurons, although astrocytic expression of mutated TDP-43 has been reported to induce neuronal cell death (Tong et al., 2013; Serio et al., 2013) suggesting that other cell types, such as microglia, are required for the neurotoxic effect. Altogether, the results indicate that astrocyte degeneration is a common feature of FTD.

## Conclusion

An increasing number of studies have connected astrocyte defects to frontal cortex pathologies. Species-specificity of astrocytes poses a challenge in translating results obtained from animal studies to humans, and patient-derived iPSCs offer an alternative to disease modeling. Studies presented above demonstrate that iPSC-derived astrocytes successfully recapitulate various disease phenotypes. Further challenges still include addressing the heterogeneity within the astrocyte population and developing protocols to generate regionally defined human astrocyte subtypes.

## Author Contributions

U-KP and MN wrote the manuscript in consultation with MC. MC performed the critical revision of the paper.

## Conflict of Interest Statement

The authors declare that the research was conducted in the absence of any commercial or financial relationships that could be construed as a potential conflict of interest.

## Abbreviations

5-HT: 5-hydroxytryptamine receptor
Aβ: amyloid β
AD: Alzheimer’s disease
APOE: apolipoprotein E
APP: amyloid precursor protein
BDNF: brain-derived neurotrophic factor
BG: Bergmann glia
cAMP: cyclic adenosine monophosphate
CTE: chronic traumatic encephalopathy
C1q: complement component 1q
EAAT: excitatory amino acid transporter
FAD: familial Alzheimer’s disease
FTD: frontotemporal dementia
GFAP: glial fibrillary acidic protein
GLAST: glutamate aspartate transporter
GLT-1: glutamate transporter 1
HD: Huntington’s disease
Htt: huntingtin
Il-1α: interleukin 1α
iPSC: induced pluripotent stem cell
JAK/STAT3: Janus kinase/signal transducers and activators of transcription 3
ROS: reactive oxygen species
SAD: sporadic Alzheimer’s disease
TNFα: tumor necrosis factor α
TDP-34: transactive response DNA-binding protein 34
TrkB: tyrosine-related kinase B
VEGF-A: vascular endothelial growth factor A
